# Intranasal Drug Delivery in Neuropharmacology: Advances in Brain-Targeted Therapies and Bioethical Challenges

**DOI:** 10.3390/biomedicines14030571

**Published:** 2026-03-02

**Authors:** Simona Irina Damian, Sofia Mihaela David, Marcela Nour, Gabriela Liliana Halitchi, Sorina Alexandra Ciurlea, Alina Stefanache, Olga-Odetta Duma, Gabriela Calin, Doina Spaiuc

**Affiliations:** 1 Grigore T. Popa University of Medicine and Pharmacy, 700115 Iasi, Romania; 2 Faculty of Medicine, “Apollonia” University of Iasi, Pacurari Str., 700399 Iasi, Romania; 3 Discipline of Pharmacology, Faculty of Pharmacy, “Vasile Goldiș” Western University of Arad, Revoluției Bv, No. 94, 310048 Arad, Romania; 4 Doctoral School of Biomedical Sciences, “Dunărea de Jos” University of Galați, 47 Domnească Street, 800008 Galați, Romania

**Keywords:** intranasal delivery, blood–brain barrier, neuropeptides, nanotechnology, bioethics, neuropharmacology, Alzheimer’s disease, oxytocin, insulin

## Abstract

Intranasal drug delivery represents a transformative “backdoor” to the brain, bypassing the blood–brain barrier (BBB) that bars 98% of small molecules and nearly all large biopharmaceuticals. By harnessing the unique anatomy of the olfactory and trigeminal nerves, therapeutics can travel directly from the nasal cavity to the central nervous system, achieving therapeutic concentrations without the systemic toxicity of traditional routes. Clinical and preclinical evidence highlight the efficacy of intranasal insulin (INI) in treating Alzheimer’s disease (AD) and delirium, with studies showing significant improvements in cognitive scores and reduced hospital stays (7.9 vs. 12.9 days; *p* = 0.014). Additionally, other peptides can be administered intranasally like oxytocin, neuropeptide Y, and novel metabolic modulators for neuroprotection and affective disorders (AD, autism, Down syndrome). Despite these promises, critical translational gaps remain, including anatomical differences between macrosmatic rodents and microsmatic humans, and significant sex-based dosing dimorphism. The ease of intranasal administration introduces profound bioethical dilemmas regarding neuroenhancement, authenticity, and informed consent in vulnerable populations. The current literature concludes that realizing the full potential of nose-to-brain (N2B) therapy requires a commitment to precision medicine, utilizing specialized delivery devices and objective biomarkers to ensure safe and equitable clinical application.

## 1. Introduction

The central nervous system is isolated from the systemic circulation by the blood–brain barrier (BBB), a highly specialized neurovascular unit composed of continuous non-fenestrated endothelial cells linked by tight junctions (*zonula occludens*), supported by a basement membrane, pericytes, and the end-feet of astrocytes. This architecture creates a physical and metabolic barrier that strictly regulates the entry of substances into the brain interstitium [1,2,3,4]. Functionally, the BBB prevents the passive diffusion of hydrophilic molecules and high-molecular-weight compounds such as proteins, peptides, and nucleic acids. Even many lipophilic small molecules are barred entry by active efflux transporters such as P-glycoprotein (P-gp) and multidrug resistance proteins. As a result, standard systemic routes of administration—oral or intravenous—often fail to achieve therapeutic concentrations in the brain without administering massive doses that can induce systemic toxicity [5,6,7]. This delivery problem is particularly acute for biopharmaceuticals like peptides, including insulin and oxytocin, which are rapidly degraded by serum proteases and eliminated by renal or hepatic clearance before they can even attempt to cross the BBB [8,9,10,11].

**Figure 1:**
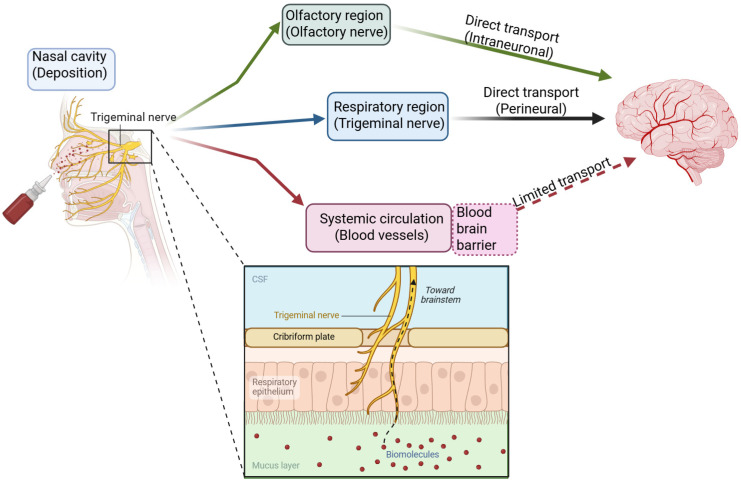
Schematic mechanisms of intranasal drug delivery. Comparison of direct nose to brain vs systemic pathways.

Intranasal administration exploits the unique anatomy of the nasal cavity, the only site in the human body where the CNS is directly exposed to the external environment (Figure 1). The nasal cavity is lined with three types of epithelium: squamous, respiratory, and olfactory. While the respiratory epithelium covering the turbinates is highly vascularized and ideal for systemic absorption, the olfactory epithelium, located at the apical region of the nasal cavity, provides a direct portal to the brain [12,13,14,15,16]. The mechanism of transport is generally tripartite, involving intracellular, extracellular, and perivascular pathways. In the olfactory nerve pathway, the olfactory epithelium contains bipolar olfactory receptor neurons (ORNs). The dendrites of these neurons extend into the nasal cavity, while their axons traverse the cribriform plate of the ethmoid bone to synapse with mitral and tufted cells in the olfactory bulb [17,18,19,20]. Therapeutics can be internalized into ORNs via endocytosis and transported intra-axonally to the olfactory bulb, a process taking hours to days. Alternatively, and more significantly for rapid drug delivery, molecules can travel extracellularly through the perineural spaces surrounding the olfactory nerve bundles. This allows direct diffusion into the cerebrospinal fluid (CSF) and the brain parenchyma, bypassing the BBB entirely [21,22].

Additionally, the trigeminal nerve (Cranial Nerve V) innervates both the respiratory and olfactory regions of the nasal mucosa. Drugs deposited in the respiratory region can enter the trigeminal nerve branches and be transported to the brainstem (pons) and other caudal structures. This pathway is particularly relevant for targeting the posterior brain and spinal cord [23,24,25,26]. Finally, the nasal mucosa is highly vascularized. While absorption into the blood vessels typically leads to systemic circulation and thus requires crossing the BBB to reach the brain, a distinct mechanism involves perivascular transport. Pulsatile arterial flow can drive the bulk flow of fluid through perivascular spaces, known as Virchow–Robin spaces, facilitating rapid transport from the nasal lamina propria into the brain. The primary advantage of intranasal delivery is its non-invasive nature, which stands in stark contrast to intracerebroventricular (ICV) or intraparenchymal injections—methods that are effective but carry significant risks of infection, tissue damage, and low patient compliance [27,28,29,30,31].

Compared to oral administration, intranasal delivery avoids the harsh acidic environment of the stomach and the extensive first-pass metabolism in the liver. This is crucial for peptide drugs like insulin or neuropeptide Y, which have negligible oral bioavailability. Nose-to-brain (N2B) delivery potentially offers a rapid onset of action [32,33,34,35,36]. Studies utilizing quantitative imaging have detected radiolabeled insulin in the brain within 30–40 min of nasal administration, a timeframe comparable to or faster than systemic distribution for some agents, without the systemic hypoglycemia associated with intravenous insulin. This pharmacokinetic profile suggests that intranasal delivery can decouple the central therapeutic effects of a drug from its peripheral side effects, a longstanding goal in neuropharmacology [37,38,39]. Despite the theoretical elegance of the N2B route, practical realization is hindered by several biological barriers. The respiratory epithelium is lined with motile cilia that beat in coordination to transport mucus toward the nasopharynx for swallowing. This mucociliary clearance mechanism clears foreign particles, including drug formulations, from the nasal cavity with a half-life of roughly 15–20 min, significantly limiting the window for absorption [40,41,42,43]. The nasal mucosa also possesses a metabolic competency lower than the liver but still significant, containing a range of metabolizing enzymes, including cytochrome P450 isoforms (CYPs), peptidases, and proteases, which can degrade peptide drugs before absorption. Finally, the nasal epithelium is tight. While more permeable than the BBB, it still restricts the paracellular transport of hydrophilic molecules larger than 1000 Daltons. Tight junctions between epithelial cells limit passive diffusion, necessitating the use of permeation enhancers or carrier systems [44,45,46,47].

A second challenge in translating N2B therapies lies in the stark anatomical differences between macrosmatic rodents and microsmatic humans. In rodents, the olfactory epithelium covers approximately 50% of the total nasal cavity surface area, providing a vast and easily accessible surface for direct neural transport. In contrast, the human olfactory region is restricted to a small niche in the superior portion of the nasal vault, accounting for only about 3% to 5% of the total surface area. The human nasal cavity’s complex geometry and longer physical distance to the cribriform plate often result in significant drug deposition in the respiratory region rather than the olfactory region, leading to higher systemic absorption and reduced brain-targeting efficiency compared to rodent models [48,49,50,51].

## 2. Advanced Formulation Strategies: Nanotechnology and Peptide Engineering

**Figure 2:**
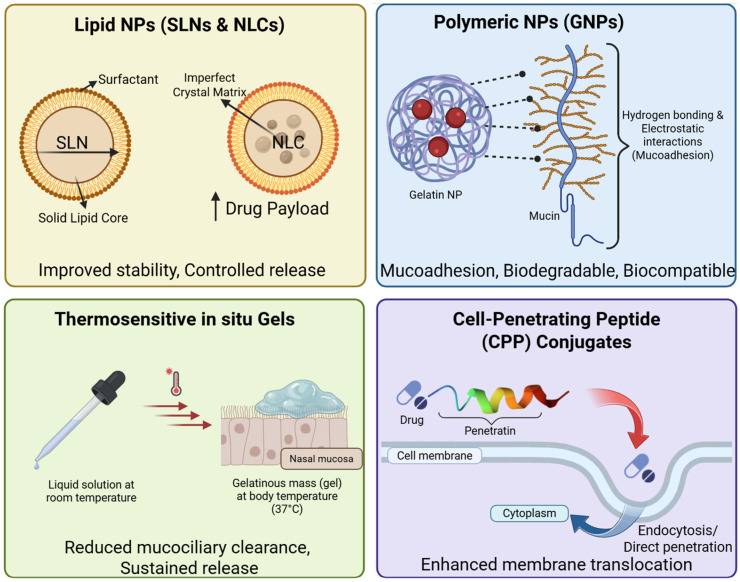
Advanced formulation strategies for intranasal delivery.

To overcome mucosal clearance and enzymatic barriers, the field has moved beyond simple saline solutions toward sophisticated nanocarrier systems and molecular conjugates. These technologies aim to protect the therapeutic payload, enhance mucosal residence time via mucoadhesion, and facilitate epithelial penetration (Table 1 and Figure 2).

Lipid nanoparticles have emerged as a dominant strategy for brain targeting due to their biocompatibility and ability to encapsulate both lipophilic and hydrophilic drugs. Solid Lipid Nanoparticles (SLNs) consist of a solid lipid core stabilized by surfactants. They offer improved stability compared to liposomes and avoid the toxicity of organic solvents used in polymeric nanoparticle production. SLNs protect encapsulated drugs from chemical and enzymatic degradation and can provide controlled release characteristics [52,53,54,55]. Nanostructured Lipid Carriers (NLCs) were developed to address the limitations of SLNs, such as low drug loading capacity and drug expulsion during storage due to lipid crystallization. NLCs incorporate a blend of solid lipids and liquid oils, creating an imperfect crystal matrix that accommodates a higher drug payload. Recent systematic reviews indicate that NLCs are increasingly preferred for neurodegenerative applications, demonstrating efficacy in delivering agents for Alzheimer’s (AD), stroke, and gliomas [56,57,58,59,60,61]. The mechanism of enhancement for lipid carriers involves both improved adhesion to the nasal mucosa and interaction with the lipid bilayer of epithelial cells, which may facilitate paracellular or transcellular passage. Additionally, surface modification of these particles with polyethylene glycol or lectins can further enhance brain targeting and reduce mucociliary clearance [62,63,64].

Polymeric nanoparticles offer versatility in surface modification and functionalization. Gelatin Nanoparticles (GNPs) are particularly notable for their mucoadhesive properties; the presence of amino and carboxyl groups allows for hydrogen bonding and electrostatic interactions with mucin, significantly prolonging nasal residence time. Studies utilizing Substance P-loaded GNPs (SP-GNPs) have shown that encapsulation protects the peptide from enzymatic degradation and enhances its uptake into the brain [65,66]. In 6-hydroxydopamine (6-OHDA) models of Parkinson’s disease, intranasal SP-GNPs demonstrated superior neuroprotection and behavioral recovery compared to SP solution, highlighting the critical role of the carrier [67,68,69]. Specifically, cell viability in SP-GNP treated groups was significantly higher than in solution-treated groups (*p* < 0.01), confirming that the carrier is essential for mediating the therapeutic effect. Analytical characterization of these GNPs reveals a mean particle size of approximately 172 nm and a negative zeta potential of −29.6 mV, properties that are ideal for avoiding rapid clearance while maintaining stability in the nasal environment [65].

Thermosensitive in situ gelling systems, such as Poloxamer-based formulations, are liquid at room temperature but undergo a phase transition to a gel upon contact with the warm nasal mucosa. This phase transition drastically reduces mucociliary clearance and provides a sustained release depot. For instance, a thermosensitive gel loaded with Dauricine was shown to increase the drug’s area under the curve (AUC) in cerebrospinal fluid (CSF) significantly compared to oral administration (*p* < 0.01), facilitating its anti-AD effects via the PI3K/AKT/mTOR pathway [70].

For large hydrophilic peptides that cannot passively diffuse across membranes, conjugation with Cell-Penetrating Peptides (CPPs) offers a solution. CPPs are short, cationic or amphipathic sequences like Tat or Penetratin capable of translocating across cell membranes via endocytosis or direct penetration. CPPs can drag their cargo across the nasal epithelium and potentially facilitate uptake into the olfactory neurons [71,72,73]. In the context of ischemic stroke, the PSD-95 inhibitor NA-1 (Nerinetide) was conjugated with Tat and LowPro peptides. This conjugation significantly enhanced NA-1 uptake into the olfactory bulb following intranasal administration, demonstrating that CPPs can effectively “ferry” therapeutics into the brain. Recent research has identified novel CPPs such as the “PLR” peptide, designed to combine membrane docking, cellular uptake, and endosomal escape functions. In vitro studies using human nasal epithelial cells showed that PLR significantly enhanced the transcytosis of both insulin and oxytocin, suggesting broad applicability [74].

**Table 1:** Comparative analysis of intranasal delivery systems.

Delivery Platform	Composition & Mechanism	Key Advantages	Major Limitations	Evidence	Type of Study	References
Standard Solution	Drug dissolved in saline/buffer; pH adjusted.	Simple manufacturing; low cost; established regulatory path.	Rapid mucociliary clearance (t_1/2_ ~15 min); high enzymatic degradation; poor bioavailability for macromolecules.	Oxytocin (standard sprays) show variable results; early Insulin trials showed limited brain uptake without devices.	Phase II/III (standard sprays); Pilot (Insulin/Oxytocin).	[75,76,77,78]
Solid Lipid Nanoparticles (SLNs)	Solid lipid core stabilized by surfactants.	Biocompatible; controlled release; avoids organic solvents; protects labile drugs.	Drug expulsion during storage (lipid crystallization); lower loading capacity than NLCs.	Effective in rodent models of AD; limited clinical translation compared to NLCs.	Preclinical (Rodent Models).	[79,80,81,82,83]
Nanostructured Lipid Carriers (NLCs)	Blend of solid lipids and liquid oils (imperfect matrix).	Higher drug loading capacity; enhanced stability; prevents drug leakage.	Complex optimization of lipid ratios; potential surfactant toxicity.	Preferred for neurodegenerative agents; high brain targeting efficiency in gliomas.	Preclinical (Animal Models).	[64,84,85,86]
Gelatin Nanoparticles (GNPs)	Cross-linked gelatin polymer.	Mucoadhesive (electrostatic interaction with mucin); biodegradable; hydrophilic core.	Batch variability of natural polymers; potential antigenicity.	Substance P: Superior recovery in 6-OHDA Parkinson’s rats vs. solution (*p* < 0.01).	Preclinical (Rat Models).	[65,87,88,89]
Thermosensitive Gels	Poloxamer or Chitosan based; sol–gel transition at 37 °C.	In situ gelling prevents runoff; drastically increases residence time.	High viscosity may impede sprayability; slower release kinetics.	Dauricine: Increased CSF AUC vs. oral (*p* < 0.01) in AD models.	Preclinical (Rat Models).	[70,90,91]
CPP Conjugates	Drug linked to Tat, Penetratin, or PLR peptides.	Facilitates intracellular uptake and transcytosis; enhances BBB/mucosal penetration.	Non-specific uptake in non-target tissues; potential toxicity of cationic peptides.	NA-1 (Nerinetide): Tat/LowPro conjugation enhanced olfactory bulb uptake.	Phase III (Nerinetide); Preclinical (Mechanism validation).	[74,92,93,94]

t_1/2_ = is the half—time of mucociliary clearance of a drug; *p* = probability value.

## 3. Therapeutic Frontiers: Metabolic Modulators and Neuroprotection

**Figure 3:**
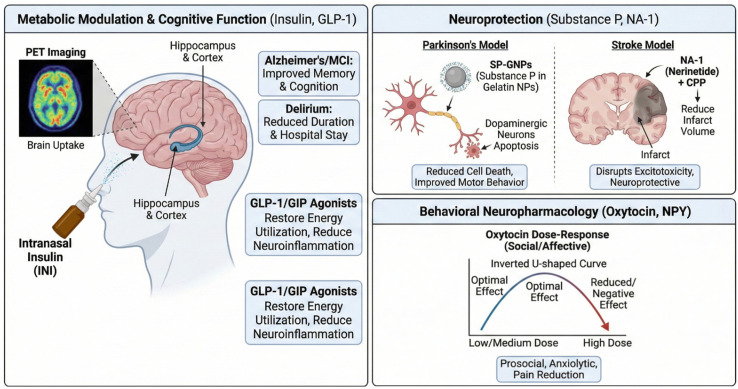
The administration of insulin, GLP-1 and neuropeptides with their specific outcomes.

The application of intranasal delivery has shown the most tangible progress in the realm of metabolic modulation and neuroprotection, driven by the strong link between brain metabolism and neurodegenerative disease (Figure 3). The hypothesis that AD represents a brain-specific form of diabetes (also called “Type 3 Diabetes” a term that is not fully supported) is supported by evidence of brain insulin resistance, reduced glucose utilization, and impaired insulin signaling pathways in AD patients. Insulin receptors are densely expressed in the hippocampus and cortex, where they mediate synaptic plasticity and memory consolidation [95,96,97,98,99].

### 3.1. Intranasal Insulin: Targeting “Type 3 Diabetes” and Delirium

Intranasal insulin (INI) has been extensively studied for its potential to restore cerebral insulin signaling without causing the systemic hypoglycemia associated with intravenous insulin. Numerous clinical trials have reported that INI improves verbal memory and cognitive function in patients with Mild Cognitive Impairment (MCI) and early AD [98,100,101]. A recent 2025 study utilizing a 2 × 2 factorial design demonstrated that 40 IU of INI, alone or in combination with the SGLT2 inhibitor empagliflozin, improved the modified Preclinical AD Cognitive Composite-5 (mPACC5) score. The combination therapy also reduced plasma GFAP, a marker of astrogliosis, and modulated biomarkers of neuroinflammation, suggesting a synergistic effect. Quantitative MRI studies using arterial spin labeling (ASL) have provided physiological validation of INI’s effects. In older adults, acute administration of INI significantly increased perfusion in the occipital gray matter (65.2 ± 11.0 vs. 61.2 ± 10.1 mL/100g/min, *p* = 0.001) and the thalamus (68.28 ± 6.75 vs. 63.31 ± 6.84 mL/100g/min, *p* = 0.003). Interestingly, in overweight men who likely possess central insulin resistance, INI decreased perfusion in limbic regions, highlighting that metabolic status fundamentally alters the brain’s hemodynamic response to insulin [38,102,103,104].

However, larger and longer trials have sometimes failed to meet their primary endpoints, often due to inconsistencies in delivery device reliability and patient status (e.g., APOE-ε4 carrier status). Currently, INI for AD is primarily in Phase II development, and large-scale, multicenter Phase III validation is still lacking [105].

A novel and highly promising application of INI is the treatment of delirium in hospitalized older adults. Delirium is metabolically characterized by acute brain insulin resistance. A randomized controlled trial published in 2025 found that long-acting intranasal insulin significantly reduced the median duration of delirium compared to placebo. Furthermore, the length of hospital stay was significantly shorter in the insulin group (7.9 days vs. 12.9 days, *p* = 0.014). This finding represents a breakthrough, offering a safe, non-sedating intervention for a condition that currently lacks effective pharmacological treatments. The field has moved beyond inferring delivery from behavioral effects. A groundbreaking PET imaging study using the radiotracer [68Ga]Ga-NOTA-insulin confirmed that intranasally administered insulin reaches key brain regions including the hippocampus, amygdala, and olfactory cortex within 30 min. The study revealed that uptake efficiency correlates with cognitive status; patients with MCI showed altered uptake kinetics compared to healthy controls, providing direct evidence that disease pathology may compromise the N2B pathway itself [38,106,107,108].

### 3.2. Incretin Mimetics and Novel Peptides

Building on the success of insulin, researchers are exploring other metabolic hormones. Glucagon-like peptide-1 (GLP-1) and Glucose-dependent insulinotropic polypeptide (GIP) agonists, widely used in diabetes, have potent neuroprotective effects. They reduce oxidative stress, normalize mitochondrial function, and dampen neuroinflammation [109,110,111,112,113]. Novel dual GLP-1/GIP receptor agonists designed to cross the BBB have shown superior efficacy in animal models of AD and Parkinson’s compared to single agonists like exendin-4 (Table 2). These agents reverse brain insulin resistance and restore energy utilization. Intranasal delivery of these peptides offers a route to maximize brain concentrations while potentially minimizing the gastrointestinal side effects common with systemic GLP-1 analogs [114,115,116].

Neuroprotective peptides such as Substance P (SP) and NA-1 (Nerinetide) are also showing promise. While classically known as a pain transmitter, SP exerts neurotrophic effects in the nigrostriatal pathway. In a rat model of Parkinson’s disease, intranasal administration of Substance P-loaded gelatin nanoparticles significantly reduced dopaminergic neuron apoptosis and improved rotational behavior. The gelatin carrier was essential for this effect, protecting SP from rapid degradation [117,118,119]. NA-1, a peptide inhibitor of PSD-95, disrupts the excitotoxic signaling cascade triggered by ischemia without blocking NMDA receptor survival signals. Intranasal delivery of NA-1, particularly when conjugated to Tat or LowPro CPPs, achieved therapeutic levels in the brain and reduced infarct volume in rodent models. Importantly, this route avoids the interactions with thrombolytic agents that have complicated intravenous trials [93,120,121].

Another significant development is the use of the KYCCSRK peptide in Down Syndrome (DS) models. DS individuals often exhibit early brain insulin resistance and AD-like pathology. Intranasal administration of KYCCSRK in Ts2Cje mice significantly reduced oxidative stress markers; 4-hydroxy-2-nonenal protein adducts (4-HNE) were reduced by 72% (*p* = 0.046) compared to untreated controls. The peptide rescued insulin signaling activation and increased mitochondrial complex levels, suggesting it targets the core metabolic dysfunctions linking DS and AD [122,123,124].

**Table 2:** Quantitative Efficacy of Intranasal Peptide Therapies in Clinical and Preclinical Models.

Therapeutic Agent	Indication/Target	Mechanism of Action	Quantitative Outcome & Significance	Type of Study	References
Insulin (40 IU)	Delirium (Human RCT)	Restores metabolic homeostasis; reduces neuroinflammation.	Hospital Length of Stay: Reduced from 12.9 days (placebo) to 7.9 days (insulin) (*p* = 0.014).	Phase III (RCT)	[38,125,126,127]
Insulin (Acute)	Aging/Perfusion (Human)	Modulates neurovascular coupling.	Occipital Perfusion: Increased to 65.2 ± 11.0 mL/100g/min vs 61.2 (placebo) (*p* = 0.001).	Phase I/II (Physiological study)	[128,129,130,131,132]
MMI-0100	Alzheimer’s (Mouse)	MK2 Inhibitor; Anti-inflammatory.	Memory Recovery: Discrimination Index restored to >60% (from chance ~50%) in Aβ/LPS models (*p* < 0.01).	Preclinical (Mouse Model)	[133,134,135,136,137,138]
KYCCSRK Peptide	Down Syndrome/AD (Mouse)	Activates Insulin Receptor & Akt; reduces BACE1.	Oxidative Stress: Reduced 4-HNE levels by 72% (*p* = 0.046) and protein carbonyls by 20% (*p* = 0.030).	Preclinical (Mouse Model)	[122,139,140]
Neuropeptide Y	PTSD/Depression (Rat)	Y1 receptor activation; Anxiolytic.	Sex-Specific Dosing: Females required 1200 μg (vs. <600 μg in males) to reduce immobility (*p* < 0.01).	Preclinical (Rat Model)	[141,142,143,144]
Dauricine (Gel)	Alzheimer’s (Rat)	PI3K/AKT/mTOR pathway modulation.	Bioavailability: AUC in CSF significantly elevated vs. oral administration (*p* < 0.01).	Preclinical (Rat Model)	[70,145,146]
Oxytocin	Post-Surgical Pain (Human)	Modulation of pain perception/anxiety.	Analgesic Request: Significantly fewer requests for pain medication in intervention group (*p* < 0.001).	Phase II/III (RCT)	[147,148,149]

AD = Alzheimer’s Disease; Akt = Protein Kinase B; AUC = Area Under the Curve; Aβ = Amyloid-beta; BACE1 = Beta-secretase 1; CSF = Cerebrospinal Fluid; 4-HNE = 4-Hydroxynonenal; IU = International Units; LPS = Lipopolysaccharide; MK2 = Mitogen-activated protein kinase-activated protein kinase 2; mTOR = Mammalian target of rapamycin; *p* = *p*-value; PI3K = Phosphoinositide 3-kinase; PTSD = Post-Traumatic Stress Disorder; RCT = Randomized Controlled Trial; μg = Micrograms.

## 4. Behavioral Neuropharmacology: Social and Affective Modulation

The intranasal route allows for the manipulation of neuropeptide systems that govern social behavior, anxiety, and memory, presenting unique opportunities for psychiatric treatment. Oxytocin (OT) is central to social cognition, trust, and bonding. Intranasal OT has been widely investigated as a treatment for the social deficits associated with Autism Spectrum Disorder (ASD) and schizophrenia. Early studies reported robust prosocial effects of intranasal OT, enhancing emotion recognition, eye gaze, and trust [150,151,152,153]. However, recent large-scale clinical trials have tempered this enthusiasm. A major randomized controlled trial evaluating high-dose intranasal OT combined with social skills training in schizophrenia found no significant benefit over placebo on primary social outcome measures. This null result highlights the complexity of translating acute behavioral effects in healthy volunteers to chronic psychiatric conditions [154].

The effects of OT are highly sensitive to context and dose. Research indicates an inverted U-shaped dose–response curve, where lower doses (e.g., 8–24 IU) may be more effective than higher doses (e.g., 40–80 IU), which can induce receptor desensitization or bind to structurally related vasopressin receptors, causing off-target effects. Furthermore, OT does not universally enhance “good” behavior; it increases in-group trust but can amplify out-group aggression or defensive responses, depending on the social context. However, in the context of pain management, results appear more consistent [155,156]. A 2025 clinical trial on post-cesarean section pain found that low-dose intranasal oxytocin significantly reduced the need for analgesic medication (*p* < 0.001) and diclofenac usage (*p* = 0.001), suggesting that its anxiolytic and modulation of pain perception pathways are clinically robust [157,158,159,160].

A primary obstacle in the clinical translation of intranasal neuropeptides like OT and NPY is the disparity between the acute, potent effects observed in preclinical models and the inconsistent results in chronic human trials. For instance, while a single intranasal dose of OT in rodents can significantly increase social preference and reduce amygdala-dependent fear expression, long-term human studies have often failed to replicate these magnitudes. In a 12-week clinical trial for autism spectrum disorder (ASD), chronic intranasal OT (16–24 IU daily) showed only modest improvements in secondary symptoms, with high placebo response rates often exceeding 30%, a phenomenon rarely captured in animal models [151,161]. Animal data suggest that chronic exposure may even lead to adverse outcomes; in male prairie voles, long-term developmental OT administration actually resulted in a 25–40% reduction in adult partner-preference formation, highlighting a potential for receptor desensitization or “inverted U-shaped” dose–response curves that are not yet fully understood in human clinical populations [162,163].

Neuropeptide Y (NPY) is an endogenous anxiolytic that regulates the stress response. In models of PTSD (Single Prolonged Stress), intranasal NPY has been shown to prevent the development of depressive-like behaviors and anxiety. A pivotal study revealed a stark sexual dimorphism in NPY dosing. While male rats responded to lower doses, females required 1200 μg of intranasal NPY to achieve the same anti-depressive effect (measured by immobility in the Forced Swim Test, *p* < 0.01) [141,164,165]. This resistance in females is attributed to higher rates of enzymatic cleavage by DPP4 or lower baseline receptor sensitivity. This finding is a critical warning for clinical trial design: “one dose fits all” approaches may doom peptide therapies to failure in female populations [142,166].

The translation of NPY findings faces similar hurdles, particularly regarding the transition from “prophylactic” animal success to “therapeutic” human application. In rodent models of PTSD, a single 100 µg intranasal NPY dose administered shortly after a stressor can prevent up to 80% of anxiety-like behaviors measured weeks later [167]. However, translating this to human chronic disorders is hindered by the fact that human patients typically present months or years after the initial trauma. The human “microsmatic” anatomy, where the olfactory region is approximately 10 times smaller relative to total nasal surface area than in rodents (3–5% vs. 50%), further limits the consistent, long-term CNS biodistribution required for chronic management [168]. Consequently, while acute animal studies provide a vital proof-of-concept, the transition to chronic human therapy requires rigorous longitudinal data to account for mucosal desensitization and the complex compensatory mechanisms of the human neuroendocrine system.

Galanin is a neuropeptide involved in depression and neurogenesis. Recent work has explored the co-administration of Galanin receptor 2 (GALR2) agonists and NPY Y1 receptor agonists. Co-administration resulted in a synergistic enhancement of spatial memory (Object-in-Place task) and neuronal precursor cell proliferation (BrdU labeling) in the dentate gyrus of the hippocampus. The effect was significantly greater than either agonist alone, suggesting that targeting receptor heteromers (GALR2/Y1R complexes) via the nasal route represents a sophisticated strategy for enhancing neuroplasticity [169,170,171]. Similarly, intranasal administration of KCNN2 blocking peptides has been shown to improve cognitive flexibility in mouse models of Fetal Alcohol Spectrum Disorders (FASD), offering a non-invasive potential treatment for developmental cognitive deficits [172].

## 5. Methodological Limitations and Translational Gaps

While the results described above are promising, a critical review reveals significant limitations that must be addressed to ensure clinical translation. The majority of successful N2B studies are conducted in rodents. However, the nasal anatomy of rodents differs fundamentally from humans. Rodents are macrosmatic; their olfactory epithelium covers approximately 50% of the nasal cavity surface area. In contrast, humans are microsmatic, with the olfactory epithelium covering only ~3–5% of the nasal surface area, located in the difficult-to-access apical crevice. This anatomical mismatch means that drug deposition efficiencies achieved in rats (often via direct instillation) are rarely replicable in humans using standard sprays. This necessitates the use of specialized delivery devices (breath-powered, precision nebulizers) in human trials to ensure the drug actually reaches the olfactory cleft [173,174,175,176].

A persistent controversy in the field is distinguishing direct N2B transport from systemic absorption that subsequently crosses the BBB. While peptides like insulin and oxytocin have poor systemic BBB permeability, small lipophilic molecules may enter the brain via both routes. In many studies, high systemic levels are detected alongside brain levels, confounding the interpretation of efficacy. Rigorous pharmacokinetic studies using radiolabeled tracers (like the [68Ga]Ga-NOTA-insulin study) are essential to quantify the exact contribution of the direct nose-to-brain fraction versus the systemic fraction [177,178,179].

The field suffers from a lack of standardization in dosing. As seen with oxytocin, dosages in trials vary widely (from 8 IU to 80 IU), leading to contradictory results. Furthermore, the reliance on behavioral endpoints (which are subjective) rather than robust biomarkers makes cross-study comparisons difficult. The recent move towards quantitative MRI (perfusion) and PET imaging represents a necessary maturation of the field, providing objective biomarkers of target engagement. Additionally, the sex differences observed in NPY dosing underscore a major gap in historical research where female subjects were often excluded; future trials must be stratified by sex to avoid false negatives [180,181,182].

Determining the optimal dose for neuropeptides in humans requires a transition from weight-based scaling to a more comprehensive model that accounts for biological sex and physiological variables. A critical challenge in intranasal delivery is that the response to neuropeptides like oxytocin and NPY is often sexually dimorphic; for instance, oxytocin has been shown to reduce sympathetic nervous system activity in women while increasing it in men during identical social stressors [183,184,185].

To standardize dosing, clinical trials should utilize Allometric Scaling based on Body Surface Area (BSA) rather than simple body weight, as BSA better correlates with metabolic rate and drug clearance across species and sexes. The *window of efficacy* for these peptides may follow an inverted U-shaped dose–response curve, where excessive doses can lead to receptor desensitization or off-target effects that differ by sex. A practical solution for clinical design is determining the Human Equivalent Dose (HED) from animal data using the FDA-recommended conversion factor (K_m_), followed by sex-stratified pilot studies to identify the specific peak of the dose–response curve for each group [186,187,188]:

$$HED\;\left(mg/kg\right)=animal\;dose\;(mg/kg)\frac{animal\;K_{m}}{human\;K_{m}}$$

- animal dose (mg/kg): the No Observed Adverse Effect Level (NOAEL);
- $K_{m}$: a constant representing the relationship between body weight (kg) and surface area (m^2^).

The FDA emphasizes a “weight-of-evidence” approach, requiring robust in vitro data on spray patterns, plume geometry, and droplet size distribution to ensure consistent delivery to the intended nasal region. Regulatory guidance (e.g., 21 CFR part 4) mandates that manufacturers evaluate the “Essential Drug Delivery Outputs” (EDDO) to guarantee that the intended dose reaches the target site accurately and reproducibly [189,190,191].

A practical solution for determining optimal human doses involves the use of objective, sex-specific biomarkers, such as functional MRI (fMRI) neural activation patterns or plasma levels of target metabolites, rather than relying on body weight alone. The *dose escalation* pilot phases in clinical trials should be stratified by sex and hormonal status (e.g., menstrual cycle phase) to establish a standardized therapeutic window that accounts for the baseline physiological differences in receptor density and sensitivity between men and women [192,193,194].

Radiotracer imaging, such as Positron Emission Tomography (PET), is frequently cited as definitive proof of N2B delivery, but these studies have significant methodological limitations that must be clarified [195]. First, spatial resolution in PET is often insufficient to distinguish between tracer accumulation in the nasal mucosa, the cribriform plate, and the superficial layers of the brain [196]. Second, many studies use tracers that are already known to cross the BBB (e.g., ^18^F-FDG), which makes it impossible to determine if the brain signal originated from the direct nasal route or from the systemic circulation [195].

Besides this, the “microsmatic” nature of the human nose compared to “macrosmatic” rodents means that tracer deposition in the small human olfactory region is often negligible, leading to poor signal-to-noise ratios. There are also radiation dosimetry concerns; the high concentration of radionuclides in the small nasal cavity can lead to unacceptably high skin radiation exposure, limiting the feasibility of these studies in healthy human volunteers [195,196].

## 6. Bioethical Challenges

The non-invasive nature of intranasal delivery, while clinically advantageous, introduces some bioethical dilemmas (Table 3). The ease of administration lowers the barrier to entry not just for patients, but for healthy individuals seeking enhancement, raising questions of safety, authenticity, and justice. Intranasal devices are user-friendly and suitable for home use. This accessibility creates a high potential for off-label use of neuropeptides as “smart drugs” or “social enhancers.” While clinical trials monitor safety in diseased populations, the long-term effects of chronic neuropeptide administration in healthy brains are unknown. There is a theoretical risk of receptor downregulation (tolerance) or the disruption of endogenous homeostatic feedback loops. For instance, chronic exogenous oxytocin could potentially impair the body’s natural production or sensitivity, leading to dependence for social functioning. The inverted U-shaped dose response of oxytocin suggests that unregulated self-administration could lead to adverse behavioral outcomes (e.g., increased anxiety or aggression) rather than the desired enhancement. The public perception of these agents as harmless “love hormones” or “brain boosters” is at odds with their complex pharmacology [197,198,199].

The ability to modulate social bonding (via oxytocin) or memory (via insulin/NPY) touches on the philosophical concept of authenticity. If a couple uses intranasal oxytocin to resolve conflict or induce bonding, is the resulting connection “authentic”? Critics might argue that chemically induced emotions lack the moral significance of those developed through interpersonal effort. If intranasal insulin becomes a known memory booster, individuals may feel compelled to use it to remain competitive. Furthermore, if these enhancements are available only to those who can afford them (or access off-label prescriptions), it exacerbates existing socioeconomic disparities, creating a “neurologically privileged” class [200,201,202,203].

The primary target demographic for many of these therapies includes patients with dementia, delirium, or severe psychiatric disorders—populations with diminished decision-making capacity. In research trials, surrogate decision-makers may conflate research participation with guaranteed clinical care (“therapeutic misconception”), driven by desperation for a cure. The non-invasive nature of a nasal spray makes it physically easier to administer to a resistant or confused patient compared to an injection. While this reduces trauma, it also lowers the threshold for overriding a patient’s refusal (non-assent) [204,205,206,207].

**Table 3:** Bioethical Frameworks and Challenges in Intranasal Neuropharmacology.

Bioethical Domain	Core Issue	Implications and Arguments	References
Off-Label Use	Unregulated consumption of “Smart Drugs” by healthy individuals.	Safety: Risk of unknown long-term side effects (receptor downregulation). Efficacy: “More is better” fallacy ignores inverted U-shaped dose response curves.	[208,209,210,211]
Neuroenhancement	Use of drugs to exceed normal species functioning (memory, sociality).	Distributive Justice: Unequal access creates socioeconomic gaps (the “neurologically privileged”). Coercion: Competitive pressure to use enhancers in academia/workforce.	[212,213,214]
Informed Consent	Research in dementia/delirium populations.	Capacity: Reliance on surrogates risks “therapeutic misconception.” Assent: Ease of nasal spray administration must not bypass patient refusal/distress.	[215,216]
Sex & Gender	Preclinical bias in dosing studies.	Justice: Historical exclusion of females in NPY studies led to ineffective dosing protocols. Need for sex-stratified trials to ensure equitable treatment efficacy.	[217,218,219,220]

## 7. Conclusions

Intranasal drug delivery has evolved from a novel anatomical curiosity to a validated, high-potential pathway for treating CNS disorders. The intranasal route has facilitated the delivery of macromolecules, such as insulin (5.8 kDa), oxytocin (1.0 kDa), and even larger proteins, that were once deemed undeliverable to the CNS due to their size and hydrophilic nature. These biopharmaceuticals are typically barred by the BBB, which restricts nearly 100% of large-molecule therapeutics. By utilizing the extracellular pathways along the olfactory and trigeminal nerves, these molecules can bypass the tight junctions of the BBB, achieving therapeutic concentrations in the cerebrospinal fluid and brain parenchyma that are unreachable via standard oral or intravenous administration. The clinical data is increasingly robust. Intranasal insulin has transitioned from experimental proof-of-concept to a demonstrated intervention for delirium and cognitive decline, supported by quantitative neuroimaging showing perfusion changes (*p* = 0.001) and reduced hospital stays (*p* = 0.014). Novel peptides like NA-1 and dual incretin agonists offer new hope for stroke and neurodegeneration, with CPPs like Tat and LowPro providing the necessary vehicle for effective brain uptake.

However, the field faces a critical juncture. The “replication crisis” in oxytocin research and the discovery of profound sex differences in peptide dosing serve as stark warnings against simplistic translation from rodent models to humans. The anatomical disparity between macrosmatic rodents and microsmatic humans remains a primary hurdle, necessitating the universal adoption of precision delivery devices in clinical trials. Furthermore, the accessibility of this technology necessitates a robust bioethical framework. As we gain the ability to easily modulate memory, mood, and social bonding via a simple nasal spray, we must rigorously define the boundaries between therapeutic restoration and enhancement. Future success will depend not only on overcoming the blood–brain barrier but also on navigating the ethical boundaries of altering the human mind. The path forward requires a commitment to precision medicine, utilizing specialized delivery devices, sex-specific dosing, and objective biomarkers, to ensure that the promise of nose-to-brain therapy is realized safely and equitably.

## Data Availability

Data sharing is not applicable to this article as no new data were created or analyzed during this study.
